# Supplementary material for: The Relationship between Food Security Status and Sleep Disturbance among Adults: A Cross-Sectional Study in an Indonesian Population
Source: Nutrients. 2020 Nov 6;12(11):3411. doi: 10.3390/nu12113411 (PMC7694601; doi:10.3390/nu12113411)
Supplement: Supplementary file 1 [file nutrients-12-03411-s001.pdf]

nutrients

Table S1. The Logistic Regression between Covariate Variables and the Outcome

Variables	Sleep Disturbance * M1		
	OR	95% CI	*p* value
Gender (ref: men)			
Women	1.17	(0.92, 1.49)	0.201
Age (years, ref: ≤ 35)			
36 - 55	1.03	(0.63, 1.67)	0.909
≥ 56	1.16	(0.50, 2.69)	0.728
Educational attainment (ref: ≥ 12 years)			
Low (< 12 years)	**0.38**	**(0.28, 0.51)**	**< 0.001**
Marital Status (ref: never married)			
Currently or Ever Married	1.43	(0.82, 2.48)	0.206
Living Areas (ref: rural)			
Urban	0.81	(0.64, 1.04)	0.096
Smoking Habit (ref: never)			
Current Smoker	0.49	(0.20, 1.16)	0.104
Quit smoker	1.06	(0.72, 1.57)	0.763
BMI (kg/m^2, ref: 18.5 - 25.0)			
25.1 - 27.0	0.90	(0.63, 1.29)	0.571
> 27.0	0.79	(0.58, 1.08)	0.135
Depressive Symptoms (ref: no)			
Yes	**3.50**	**(2.72, 4.52)**	**< 0.001**
Food Security Status (ref: food secure)			
Food Insecure	**1.47**	**(1.15, 1.89)**	**0.002**

Abbreviations: OR, Odd Ratio; CI, 95% confidence interval; M1, Model 1. Note: * Sleep disturbance was defined as greater than mild or without greater than mild. Models are adjusted for age, gender, body mass index, education attainment, marital status, living areas, smoking habits, physical activity volumes, blood pressure values, and food consumption score, CES-D-10 Score. Model 1 is adjusted for age and gender. All statistically significant values are set to < 0.05.

Table S2. The Logistic Regression between Characteristics related to The Sleep Disturbance Level Stratified by Gender.

Variables	Sleep Disturbance (Men)			Sleep Disturbance (Women)		
	OR	95% CI	p value	OR	95% CI	p value
Age (years, ref: ≤ 35)						
36 - 55	1.04	(0.68, 1.57)	0.866	1.73	(1.00, 1.82)	0.016
≥ 56	1.26	(0.65, 2.40)	0.493	2.54	(1.17, 2.72)	0.002
Educational attainment (ref: ≥ 12 years)						
Low (< 12 years)	0.46	(0.30, 0.71)	< 0.001	0.37	(0.22, 0.61)	< 0.001
Marital Status (ref: never married)						
Currently or Ever Married	1.64	(0.82, 3.25)	0.158	1.61	(0.57, 4.55)	0.374
Living Areas (ref: rural)						
Urban	1.13	(0.78, 1.65)	0.526	0.88	(0.62, 1.25)	0.483
Smoking Habit (ref: never)						
Current Smoker	0.35	(0.12, 0.99)	0.048	2.12	(0.47, 9.55)	0.329
Quit smoker	0.78	(0.50, 1.20)	0.251	0.78	(0.30, 2.02)	0.605
BMI (kg/m2, ref: 18.5 - 25.0)						
25.1 - 27.0	1.50	(0.90, 2.49)	0.121	0.66	(0.40, 1.11)	0.118
> 27.0	0.90	(0.50, 1.63)	0.732	0.77	(0.52, 1.15)	0.201
Depressive Symptoms (ref: no)						
Yes	3.16	(2.17, 4.60)	< 0.001	3.93	(2.77, 5.57)	< 0.001
Food Security Status (ref: food secure)						
Food Insecure	1.75	(1.19, 2.58)	0.005	1.07	(0.76, 1.51)	0.706

Abbreviations: OR, Odd Ratio; CI, 95% confidence interval. Note: * Sleep disturbance was defined as greater than mild or without greater than mild. Models are adjusted for age, gender, body mass index, education attainment, marital status, living areas, smoking habits, physical activity volumes, blood pressure values, and food consumption score, CES-D-10 Score. All statistically significant values are set to < 0.05.

nutrients MDPI

Table S 3. The Regression Test between Depressive symptoms and Sleep Disturbance by Food Security Status.

Variable			Model 1			Model 2			Model 3		
	Dependent	Independent	OR	95% CI	*p* value	OR	95% CI	*p* value	OR	95% CI	*p* value
Food Secure	Sleep Disturbance *	Depressive Symptoms	2.99	(1.97, 4.52)	< 0.001	3.34	(2.19, 5.09)	< 0.001	3.22	(1.26, 8.24)	0.015
Food Insecure	Sleep Disturbance *	Depressive Symptoms	3.08	(2.25 - 4.21)	< 0.001	3.67	(2.67 - 5.05)	< 0.001	3.18	(1.61 - 6.28)	0.001
			β	95% CI	*p* value	β	95% CI	*p* value	β	95% CI	*p* value
Food Secure	Sleep Disturbance Score	CES-D-10 Score	2.33	(2.23, 2.43)	< 0.001	2.32	(2.22, 2.43)	< 0.001	2.25	(2.04, 2.48)	< 0.001
Food Insecure	Sleep Disturbance Score	CES-D-10 Score	2.32	(2.23, 2.42)	< 0.001	2.32	(2.23, 2.42)	< 0.001	2.17	(1.97, 2.39)	< 0.001

Abbreviations: OR, Odd Ratio; CI, 95% confidence interval; β, exponentiated beta coefficient; FCG, food consumption group. Note: * Sleep disturbance was defined as greater than mild or without greater than mild. Model 1 was a unadjusted model. Model 2 was a model with adjustment for age and gender. Model 3 was a Model 2 + adjustment for body mass index, education attainment, marital status, living areas, the status of smoking habit, physical activity volumes, blood pressure values, ethnicity, and chronic diseases (e.g., diabetes or cardiovascular diseases). All statistically significant values are set to < 0.05.

nutrients MDPI

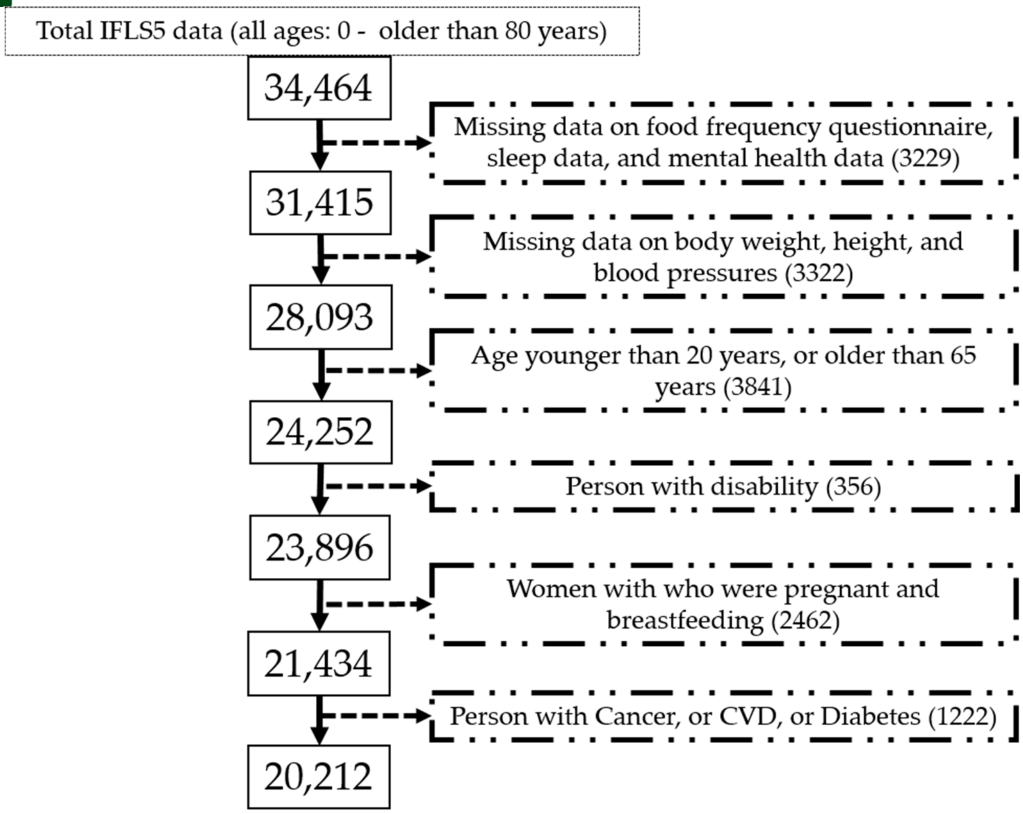

Figure S 1. Flowchart of the Sampling Participants

© 2020 by the authors. Submitted for possible open access publication under the terms and conditions of the Creative Commons Attribution (CC BY) license (http://creativecommons.org/licenses/by/4.0/).
